# Supplementary material for: Change of intestinal microbiota in mice model of bronchopulmonary dysplasia
Source: PeerJ. 2022 Apr 20;10:e13295. doi: 10.7717/peerj.13295 (PMC9034698; doi:10.7717/peerj.13295)
Supplement: Supplemental Information 1 [file peerj-10-13295-s001.docx]

Supplementary Table 1. The statistics in the process of processing reads

| Day | Group | Raw Reads | Clean Reads | Effective Reads | Average length(bp) | GC(%) | Effective(%) |
| --- | --- | --- | --- | --- | --- | --- | --- |
| 7d | Control | 76857.6±6600 | 75792.8±6522 | 72672.8±7687 | 421.8±2.04 | 53.228±0.692 | 94.384±2.583 |
|  | BPD | 80072.4±221 | 79003.8±212 | 77170.2±445 | 424±0.63 | 52.934±0.438 | 96.374±0.379 |
| 14d | Control | 70381±11959 | 69371.6±11797 | 68194.6±11464 | 425.2±0.4 | 49.966±0.999 | 96.928±0.39 |
|  | BPD | 80005.6±190 | 78893.2±204 | 77605.2±265 | 425.6±1.36 | 52.538±0.562 | 96.998±0.196 |
| 21d | Control | 74138.8±11806 | 73100.6±11643 | 71860.6±11445 | 421±2.19 | 52.982±0.366 | 96.928±0.046 |
|  | BPD | 80108.4±168 | 79028.4±182 | 77388±850 | 421.2±1.33 | 53.302±0.27 | 96.604±0.939 |

Raw reads is the number of original reads obtained by sequencing; Clean reads is the number of high-quality reads obtained after quality control and splicing of the original sequence; Effective reads is the number of effective sequences filtered by clean reads; Average length is the average sequence length of the sample; GC (%) is the percentage of G and C type bases in the total bases; Effective (%) is the percentage of effective reads in raw reads.
