# Supplementary material for: Change of intestinal microbiota in mice model of bronchopulmonary dysplasia
Source: PeerJ. 2022 Apr 20;10:e13295. doi: 10.7717/peerj.13295 (PMC9034698; doi:10.7717/peerj.13295)
Supplement: Supplemental Information 2 [file peerj-10-13295-s002.docx]

Supplementary Table 2. Metastats on phylum level between two groups on day 14

| **Variable** | **Control** | **BPD** | **Variation** | **P_FDR_** |
| --- | --- | --- | --- | --- |
| *Cyanobacteria* | 6.20E-04±1.08E-04 | 6.03E-03±9.29E-04 | Increase | 0.00E+00 |
| *Bacteroidetes* | 5.48E-01±7.26E-02 | 1.16E-01±4.85E-02 | Decrease | 3.83E-03 |
| *Acidobacteria* | 1.93E-03±5.58E-04 | 7.60E-03±1.01E-03 | Increase | 7.33E-03 |
| *Chloroflexi* | 3.45E-04±7.75E-05 | 1.21E-03±1.76E-04 | Increase | 8.61E-03 |
| *Rokubacteria* | 6.74E-05±2.17E-05 | 4.23E-04±7.94E-05 | Increase | 8.61E-03 |
| *Epsilonbacteraeota* | 2.16E-03±6.32E-04 | 1.78E-02±3.81E-03 | Increase | 1.15E-02 |
| *Proteobacteria* | 5.14E-02±1.02E-02 | 2.98E-01±6.34E-02 | Increase | 1.18E-02 |
| *Nitrospirae* | 1.33E-04±5.54E-05 | 4.60E-04±7.33E-05 | Increase | 1.71E-02 |
| *Gemmatimonadetes* | 1.35E-04±5.26E-05 | 6.63E-04±1.43E-04 | Increase | 1.75E-02 |
